# 675 Reducing Hospital-Acquired Pneumonias in Pediatric Burn Care: A Multidisciplinary Approach

**DOI:** 10.1093/jbcr/iraf019.304

**Published:** 2025-04-01

**Authors:** Vickie Walker, Peggy Glanders

**Affiliations:** Shriners Children’s - Texas; Shriners Children’s - Texas

## Abstract

**Introduction:**

Hospital-acquired pneumonia (HAP) is a significant concern in pediatric burn care facilities, impacting patient outcomes and healthcare costs. In our facility, the incidence of HAP was 1.07 per 1,000 patient days in 2020 (4 HAPs/3749 patient days). Remarkably, there were no HAP cases in 2021. However, in 2022 the incidence rose to 1.29 per 1,000 patient days (5 HAPs/3866 patient days). This fluctuation emphasized the need for a systematic approach to prevent HAPs and maintain patient safety.

**Methods:**

A multidisciplinary team comprising Respiratory Therapy, Therapy Services (Speech, Occupational Therapy, Physical Therapy), Infection Control, Nursing Services, Nutritional Services, and Pharmacy monitored the implementation of the pneumonia prevention bundle. The team observed 51 patients ranging from infancy to 17 years old. The following bundle items were monitored: elevated head of bed (HOB), daily sedation vacation and assessment of readiness to extubate, peptic ulcer disease prophylaxis, deep venous thrombosis (DVT) prophylaxis in children > 10 years of age, oral care with an antiseptic and disinfectant, subglottic suctioning, nutritional support, early mobilization, and hand hygiene compliance. Specific assessment tools ensured comprehensive implementation and evaluation.

**Results:**

In 2023, the implementation of the pneumonia prevention bundle resulted in 0 HAP cases over 3809 patient days, achieving a significant reduction in the incidence of HAPs.

**Conclusions:**

The successful implementation of the pneumonia prevention bundle led to the elimination of HAP cases in 2023. This outcome underscores the effectiveness of a multidisciplinary approach and systematic prevention strategies in enhancing patient safety and optimizing care delivery in pediatric burn care settings.

**Applicability of Research to Practice:**

The findings advocate for the continued use and potential adaptation of the bundle to maintain low HAP rates and improve overall patient outcomes.

**Funding for the Study:**

N/A

